# The development of Tobacco Harm Prevention Law in Vietnam: stakeholder tensions over tobacco control legislation in a state owned industry

**DOI:** 10.1186/1747-597X-6-24

**Published:** 2011-09-18

**Authors:** Hideki Higashi, Tuan A Khuong, Anh D Ngo, Peter S Hill

**Affiliations:** 1 The University of Queensland, School of Population Health, Herston, QLD 4006, Australia; 2 Health Strategy and Policy Institute, Hanoi, Vietnam; 3 Vietnam Evidence for Health Policy Project, School of Population Health, University of Queensland, Hanoi, Vietnam; 4 Social Epidemiology and Evaluation Research Group, School of Health Sciences, University of South Australia, Adelaide, SA 5000, Australia

## Abstract

**Background:**

Building on its National Tobacco Control Policy initiated in 2000, Vietnam is currently considering introducing a comprehensive law to strengthen the implementation of tobacco control policy. This study analyses the positions of key stakeholders in the development of tobacco control legislation in the context of a largely state-owned industry, and discusses their implications for the policy process.

**Methods:**

Several qualitative methods were employed for the study including: literature review and documentary analysis; key informant interview; focus groups discussion; and key stakeholders survey.

**Findings:**

The Ministry of Health, Ministry of Trade and Industry, and Ministry of Finance are key players in the tobacco control policy and legislation, representing competing bureaucratic interests over health, macro-economy and revenue. High-ranking officials, including the Communist Party and National Assembly members, take a rather relaxed position reflecting the low political stakes placed on tobacco issues. The state-owned tobacco industry is regarded as an important contributor to the government revenue and gross domestic product, and the relative weight on health and socioeconomic issues placed by stakeholders determine their positions on tobacco control. Overall, short-term economic interests have more immediate influence in setting policy directions, with the consequences of health gains perceived as relegated to a distant future. This was reflected in the position of tobacco control advocates, including MOH, that presented with reluctance in insisting on some tobacco control strategies revealing a mixture attitude of concessions to the socioeconomic uncertainties and a sense of bargaining to win the strategies that are more likely to be accepted.

**Conclusion:**

The state-ownership of tobacco industry poses a major paradox within the government that benefits from manufacturing of tobacco products and is also responsible for controlling tobacco consumption. The perceptions of negative implications on government revenue and the macro-economy, coupled with the reluctance to challenge these issues from health perspective too directly, means that tobacco control has yet to secure itself a place on the priority policy agenda. The overall policy environment will shift in favour of tobacco control only if the economic framing can be challenged.

## Background

Vietnam is an emerging middle-income country in Southeast Asia with a population of over 86 million [1], having shifted from a centrally planned economy towards a more market-oriented economy since the inception of *Doi Moi *(renovation) policy in 1986. The reforms have brought remarkable economic growth with an increase of gross domestic product (GDP) per capita from USD 400 in 2000 to USD 1,160 in 2010 [2]. Despite this economic liberalisation, Vietnam has maintained a socialist political system, with the state assuming central role in economic development. Major enterprises, including tobacco, remain largely state-owned [3].

Parallel to its economic growth, population disease patterns are changing in Vietnam, with a decline in communicable diseases, increasingly replaced by non-communicable diseases over recent years [4]. Life-style related health risks are becoming increasingly recognised as important. High among these risk factors is tobacco consumption: Vietnam has one of the highest smoking rates in the world. Findings from national surveys suggest rates of smoking among male adults in excess of 50% [5-7], though smoking is not a common practice among females of whom less than 5% smoke. In August 2000, the government of Vietnam issued a resolution on the National Tobacco Control Policy 2000-2010 [8]. The resolution was a manifestation of the government's commitment to combat the epidemic of tobacco related diseases in Vietnam, anticipating its political participation in the upcoming Framework Convention on Tobacco Control (FCTC). The former Steering Committee on Tobacco Control under the Ministry of Health (MOH) was upgraded in 2001 to become the Vietnam Steering Committee on Smoking and Health (VINACOSH) that currently involves five ministries and five mass organisations (largely affiliated with the Communist Party, such as the Vietnam Fatherland Front) as its members. On 21 May, 2003, Vietnam was represented in the World Health Assembly when it adopted the FCTC, and in 2004, was one of the first nations in the region to ratify the FCTC. The action plan to implement FCTC was launched in August 2009 [9], and the new Tobacco Harm Prevention Law is being drafted at the time of this research [10].

Since the inception of the National Tobacco Control Policy, over fifteen decrees and decisions have been issued for implementation, including an increase of excise tax and smoking ban in designated public places. Educational messages appear on the media regularly, and warning labels on cigarette packages have been enlarged and brought to the front panel with clearer messages on the associated risk of smoking-related diseases. The proposed Tobacco Harm Prevention Law includes a set of regulations on tobacco manufacturing, trade and distribution, smoke-free environment and protection from exposure to second-hand smoke, restriction on tobacco image use in art products, smoking cessation activities, and a tobacco control fund. Yet, despite the apparent vigour with which the government of Vietnam has responded to the international agenda on tobacco control, progress with legislation and implementation has been cautious.

This study aims to analyse the positions of key stakeholders in the development of Tobacco Harm Prevention Law in the context of a largely state-owned industry in Vietnam, and discusses their implications for the policy process, and the tensions that cause the paradox between the government commitment to tobacco control and delays in finalising and passing the Tobacco Harm Prevention Law.

## Methods

This study employs a case study approach [11] adapting Varvasovszky and Brugha's stakeholder analysis guidelines to identify and analyse the positions of key stakeholders, their primary interests and power relationships, and their engagement with the proposed Tobacco Harm Prevention Law [12]. In terms of an explanatory model for examining differing stakeholder perspectives on policy, we use Grindle and Thomas' bipolar characterisation of policy as either "crisis"--those issues perceived as requiring urgent, decisive action to ensure political stability--or "politics-as-usual"--those more routine policy issues that do not generate the same sense of priority, and may be engaged over a longer time frame [13].

Despite the recent economic changes implicit in *Doi Moi*, Vietnam maintains a one-party dominated socialist political system, and as in all "elite" interviewing [14], access to some policy stakeholders can be difficult [15]. To increase our rigour in seeking insight into the positions of relevant stakeholders, and to address their complex political agendas, we have employed a multi-strategic approach in this research, triangulating findings from both direct and indirect sources and complementary research methods [16].

The research was undertaken through literature review and documentary analysis, key informant interviews and a focus group discussion, and a survey of selected members of the National Assembly. The literature review explored tobacco control policy, policy analysis, the evidence on available tobacco control interventions, and the political system with reference to Vietnam, other communist states and developing countries.

Electronic literature searches of PubMed, Medline, Google and Google Scholar were performed with key words including "tobacco control", "policy analysis", "Vietnam", "communist state", "developing country", "political system".

Additionally, government reports, donor reports and legislative documents on the political context, and tobacco smoking in Vietnam, were obtained with assistance of the Health Strategy and Policy Institute (MOH) for analysis. The analysis focused on potential stakeholders involved in tobacco control policy, the magnitude of tobacco related disease burden, and the progress of the formulation and implementation of tobacco control policies in Vietnam.

**Figure 1:**
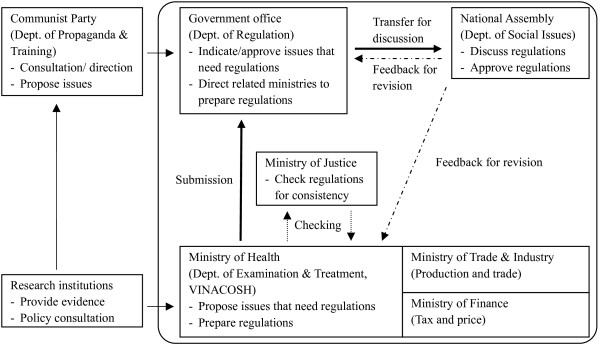
**Legislation process of Tobacco Harm Prevention Law**.

In consultation with VINACOSH, key informants were identified using an "event-based" sampling approach [17,18] based on the legislation process for the Tobacco Harm Prevention Law. Three major ministries were identified as key actors in tobacco control policy: Ministry of Trade and Industry (MTI); MOH; and Ministry of Finance (MOF) (see Figure 1).

In addition, seven critical actors were identified across different sectors and hierarchical levels for interview, based on their roles in the policy process (Table 1). Despite introductions through VINACOSH, responsible for liaising with other ministries on the Tobacco Harm Prevention Law, informants from the state-owned Vietnam National Tobacco Corporation (VINATABA) declined to be interviewed. The interviews were semi-structured, aimed at exploring the role of each stakeholder and their position in the development of Tobacco Harm Prevention Law, respondents' perceptions of proposed policy instruments, and the tensions between stakeholders with competing agendas. Interviews were completed between February and March 2010 (HH, TAH, ADN) in English or in Vietnamese assisted by an interpreter. The interviews were pretested with staff from the Health Strategy and Policy Institute.

**Table 1:** Stakeholders included in the key informant interviews

Stakeholder	Represented interests
National Assembly Office (*NAO*)	Legislative branch
Government Office (*GO*)	Executive branch
Department of Propaganda and Training, Communist Party (*DPT*)	Party
Bureau of Treatment and Examination, MOH (*MOH-TE*)	Ministry (public health)
Bureau of Legislation, MOH (*MOH-L*)	Ministry (health-related law)
Vietnam Steering Committee on Smoking and Health (*VINACOSH*)	Ministry (tobacco control)
Ministry of Trade and Industry (*MTI*)	Ministry (industry, economics)
Ministry of Finance (*MOF*)	Ministry (revenue, budget)
Vietnam Public Health Association (*VPHA*)	Academic public health, civil society
World Health Organization (*WHO*)	Multilateral agency
HealthBridge Canada (*HBC*)	International NGO

NB: The abbreviations in Italics represent the informants in the interviews and focus group discussion, and denote quotes from the interviewees in the text.

In addition, one 60-minutes focus group discussion was held with six staff members of the MOH aimed to further elaborate on the perceptions on the strategies, potential obstacles, and opportunities to progress with the Tobacco Harm Prevention Law.

All interviews and discussions were transcribed into Vietnamese or English, depending on the language used for each session, and were summarised in English. Thematic analyses were conducted on textual data, assisted by the ATLAS.ti 6.1 programme [19]. Emergent themes were discussed by all researchers for a final consensus. Ethics clearance was obtained from the School of Population Health Research Ethics Committee at the University of Queensland.

In May and November 2009, VINACOSH was invited by the National Assembly to provide information sessions on the Tobacco Harm Prevention Law to members of the National Assembly and their staff in Hanoi and in one Northern Province. A self-administered questionnaire was distributed as part of the evaluation process linked to these information sessions, providing an exceptional opportunity to access the perceptions on tobacco control of difficult-to-access higher-ranking officials. Response rates were 67 (65%) and 35 (50%) for the first and second sessions, respectively (Table 2). The central session conducted in Hanoi was dominated by central level policy officers and administrative staff, with a proportionately higher representation of provincial level officers and health professionals at the provincial session. The questionnaire comprised structured and semi-structured questions related to tobacco control policy and the proposed legislation including: overall understanding of tobacco as a public health issue; perceptions on the available evidence surrounding tobacco control activities; and positions to the proposed tobacco control legislation (see Additional file 1: Questionnaire form).

**Table 2:** Composition of survey respondents at the National Assembly

Category	**1**^**st **^**survey**	**2**^**nd **^**survey**
**Administrative level**		
Central	34 (50.8)	8 (22.9)
Provincial	21 (31.3)	22 (62.8)
District	1 (1.5)	2 (5.7)
Commune	0 (0.0)	0 (0.0)
Missing	11 (16.4)	3 (8.6)
**Total**	67 (100)	35 (100)
		
**Occupation**		
Medical doctor	9 (13.4)	11 (31.4)
Teacher	1 (1.5)	0 (0.0)
Reporter	1 (1.5)	0 (0.0)
Manager	4 (6.0)	7 (20.0)
Bonze	1 (1.5)	0 (0.0)
Officer	15 (22.4)	4 (11.4)
Other	16 (23.9)	5 (14.3)
Missing	20 (29.8)	8 (22.9)
**Total**	67 (100)	35 (100)

Numbers in parentheses represent percentages.

## Results

### Key policy decision makers in Vietnam

#### The Communist Party of Vietnam

The political system in Vietnam is highly centralised and represents a "mono-organizational socialism" in which the Communist Party exercises sole power as stated in the Vietnamese Constitution. The Party takes overall leadership on all aspects of politics and state administration [15]. As one of the informants succinctly described: "the Party directs, the government manages, and the ministries follow" (*DPT*; quote from interview).

The ongoing legitimacy of Party control has been maintained by multiple factors. Using Thayer's [20] adaptation of Weber's classification [21], the Party gains charismatic legitimacy from the legacy of Ho Chi Minh; performance legitimacy through its high economic growth since *Doi Moi*; rational-legal legitimacy through the 1992 Constitution [22]; and international legitimacy through its membership in the Association of Southeast Asian Nations (ASEAN), World Trade Organization (WTO) and others.

However, although there has been a growing separation of Party and state since the introduction of the *Doi Moi *policy, the Party maintains its direct influence on state activities such as the careers of senior officials [23], licensure for religious activity [24], active control of political dissent [20] and the retention of certain policy positions such as the one-to-two child family [25]. More importantly, the Party maintains a shadow structure that runs parallel to all political institutions through which it exerts its authority by manipulating the processes in the formal structure [26]. The Party General Secretary is one of highest figures amongst the leadership in Vietnam, with the majority of senior positions in the government filled by Party members. Party influence is extensive throughout the policy decision-making process prior to its introduction to the National Assembly, though elements of the Party such as its Department of Propaganda and Training, would only become involved once the policy had been determined (*DPT*; interview).

With overarching responsibilities for both economic and social control, however, the Party presented an ambivalent position on tobacco control policy, with the respondent's major concern identified as how to offset the imperatives of macroeconomic development against the health issues associated with tobacco:

"... we must keep the balance between production of tobacco, distribution of tobacco products, and promotion of public health." (*DPT*; quote from interview)

#### The National Assembly

The National Assembly is the legislative body of Vietnam. Constitutionally, the Assembly "is the highest representative organ of the people and the highest organ of State power of the Socialist Republic of Vietnam" [22]. The Assembly has the power to prepare, adopt and amend the Constitution as well as making laws, and to implement state plans and budgets. However, its primary function is to concretise the Party's decisions into laws and decrees, with the final approval or annulment rights retained by the Party [27]. Similar to the senior positions at the government, the majority of seats at the Assembly are filled by members from the Party, although a few are conceded to approved "independents". A general national election is held every five years at which people vote from candidates pre-screened by the Party.

Although the legislation process is a closed process in principle, it is gradually opening up to accommodate the ideas and opinions of the civil society and general public:

"... the National Assembly has a mechanism to open the floor to non-governmental organisations and public to comment on that [draft law]. ... I think Vietnam is gradually opening up to the public. For example, we are invited to the National Assembly sessions. I think it is something very advanced in Vietnam. They openly talk to us; they encourage us to attend to give our comments." (*HBC*; quote from interview)

However, tobacco control has yet to secure itself a place on the priority agenda for most Assembly members. From the results of the National Assembly survey, fewer than half the respondents recognised the scale of tobacco-related mortality as larger than the combined mortality caused by HIV/AIDS, malaria and tuberculosis. Surprisingly, the respondents in the first session, which was dominated by central and Provincial administrators and bureaucrats, had better ideas on the issues surrounding tobacco and were more supportive of control, while those in the second, Provincial session--where close to one third of the National Assembly representatives and staff were medical officers--seemed to be less informed (see Table 2). There are two potential implications: firstly that the tobacco control agenda has not been communicated strongly through the medical profession (35% of health professionals smoke [28]), and secondly, decisions on tobacco control made at the central level may lack the appropriate perception of political priority for implementation at local levels.

Again, economic considerations were prominent in the National Assembly responses:

"To convince the National Assembly and the government of the proposed Tobacco Harm Prevention Law, MOH must show evidence on tobacco harms, and the perceived impact on the society and economy." (*NAO*; quote from interview)

#### The government and affiliated bodies

The primary role of the government is to translate the Party's directives into strategies and plans for implementation [15] through its 16 ministries and 5 ministry-level organisations. The duties of the government includes drafting laws, decrees, state plans and budgets, as well as their subsequent implementation upon approval; assuring national security; organising foreign relationships; and economic development [27]. Ministries are at the forefront of government bureaucracy, preparing strategies and development plans, drafting policies, laws and regulations, and adopting regulatory documents for policy implementations [29]. The structure of ministries is similar, with the office of the Minister, professional and functional divisions, training centres, state enterprises and research institutes relevant to the field. The decision for recruitment of civil servants in the central ministries is directly made by the Ministers, based on the recommendations from the Director of Personnel, and senior positions are, almost without exception, filled by Party members [29].

Although five ministries and five mass organisations are involved in VINACOSH, three major ministries prevail as key actors in tobacco control policy: MTI, MOH, and MOF, and their stated positions reflect the complexity of the issues.

The Ministry of Health maintains the strongest position on tobacco control policy and law. Its mandate to ensure public health is the driving force in the effort to reduce tobacco consumption among the Vietnamese population. Headed by the Minister of Health, though multi-sectoral in nature, VINACOSH coordinates tobacco control activities, and has been responsible for developing the Tobacco Harm Prevention Law.

The Ministry of Trade and Industry, which controls VINATABA and as a consequence has a clear vested interest in resisting tobacco control initiatives, claims to be cooperative in the effort to reduce smoking-related harms. Although MTI does not necessarily see tobacco as a priority industry for further investments and production growth, as clearly proscribed by the National Tobacco Control Policy [30], it is still an important contributor to the GDP and tax revenue. In its response, MTI argues that the legislation proposed by the MOH is too narrow in perspective:

[The Tobacco Harm Prevention Law] "is one-sided from the perspective of health sector only, primarily focusing on the reduction of supply. The MOH proposed law should be part of a comprehensive tobacco control law that is harmonised with different stages of tobacco industry such as production, manufacturing, and trade." (*MTI*; quote from interview)

The Ministry of Finance stands between these two ministries, responsible for collecting tax from industry and allocating budget to different sectors, including health. However, interviews with MOF officials suggest that the economic and financial perspectives of MOF currently align it more closely with MTI than MOH. Clearly, as Miles' Law points out: "Where you stand depends on where you sit" [31]. The position of MOF reflects the relative importance of the industry, and its dominance over the other social sectors.

"Tobacco industry contributes 15% of the government revenue. ...a high excise tax will increase smuggling that is still out of control. ...We should have a reasonable strategy for excise tax. Otherwise, it will affect the socioeconomic development and result in increased smuggling." (*MOF*; quote from interview)

"MTI is very important, MOF is controlling the budget, and certainly both have powerful voices in the government, while MOH is less powerful [since it] is related to health issues only." (*WHO*; quote from interview)

The Government Office is responsible for receiving and approving (or annulling) draft laws proposed by all ministries and submit to the National Assembly for initial review. Once it receives feedback from the Assembly, the Government Office coordinates with relevant ministries to finalise the draft law for and formally introduce to the Assembly session for ratification. Even after the draft law has been fed into the formal process, the Government Office is still in a position to coordinate with the relevant committees at the National Assembly.

Representatives of the Government Office presented a neutral position to the content of the Tobacco Harm Prevention Law, reflecting its role as a coordinating body of all sectors. Nonetheless, its power to prioritise which draft laws it introduces to the National Assembly, means its position in relation to the Tobacco Harm Prevention Law has important implications.

### Tobacco control advocates

The Tobacco Control Working Group assumes a central position in coordinating tobacco control advocates in Vietnam. Convened in 2003 by the World Health Organization (WHO) and HealthBridge Canada, it has grown rapidly which now involves over 70 members from various organisations at different levels including government agencies, international agencies, civil society organisations and academia [32].

International agencies from bilateral and multilateral donors can be powerful in influencing the decision-making process of a host government [13]. However, few international stakeholders are involved in the tobacco control dialogue in Vietnam. There are only two UN and five international non-governmental organisations (NGOs) involved in the Tobacco Control Working Group [32]. Some respondents from the interviews attributed the slow process of tobacco control policy to the lack of international pressure. This is in contrast to the HIV/AIDS policy which was endowed with major international support for its technical aspects, resource mobilisation, and in influencing the opinions of high-level decision makers [15], and in the word of one respondent, putting "strong pressure to the government" (*VINACOSH*; quote from interview).

The primary roles of WHO, HealthBridge Canada, Atlantic Philanthropies and others, which are involved in the Tobacco Control Working Group, are to provide technical and financial support to facilitate tobacco control without exerting substantive political influence on the government. With the Japanese government and the World Bank, the largest bilateral and multilateral donors in Vietnam, not engaged in this arena, the policy leverage of international actors is relatively low in tobacco control.

Several civil society organisations are engaged in tobacco control activities, with ten local NGOs currently involved in the Tobacco Control Working Group [32]. Different NGOs have various focuses in tobacco control which may include pilot projects (e.g. smoke-free community, quit-line), health education, advocacy and research. However, these NGOs have mostly emerged since the adoption of *Doi Moi *in the 1980s, with the evolution of civil society still in its early phase. The post *Doi Moi *influx of foreign aid, including international NGOs, has catalysed a new paradigm within civil society. Many international NGOs rushed into Vietnam with models that provide aid through counterpart NGOs [33]. These were meant to pursue a bottom up approach to partner with "local" NGOs, with an emphasis on participatory development. This transition has triggered an explosion in the number of civil society activities throughout the 1990s, and by 2005, 140,000 community-based organisations, 3,000 cooperatives, 1,000 locally registered NGOs and 200 charities were present in Vietnam [33]. However, many local NGOs view themselves as a partner to, or an extension of, the state-run mass organisations to support existing government policies, and most, if not funded by the government, are heavily regulated by it [33]. Therefore civil society operates, in a sense, within the constraints of the one-party framework.

The Vietnam Standard and Consumer Association (VINASTS), for instance, is actively involved in the Tobacco Control Working Group representing civil society. Its commitment in tobacco control features policy advocacy and public education [32]. However, its founding members comprised former Party executives and officials, and senior positions have been held by retired officials from various ministries or state-owned enterprises [34]. The Vietnam Public Health Association (VPHA) is a social and professional association, which now assumes the leading role in the Tobacco Control Working Group. VPHA's main function is evidence-based advocacy for new policies or implementation of existing policies, including tobacco control, through pilot projects and research. Similar to other local NGOs, it is financially managed by MOF and was admitted as a member of the Vietnamese Fatherland Front, the "political base of people's power" [22]. But the unique position of VPHA, as a result of these connections, is its capacity to link tobacco control advocates with high-ranking policy makers, giving them channels to "access and approach the National Assembly members" (*VPHA*; quote from interview). While its position differs from typical NGO relationships with government as understood in Western contexts, it is invaluable for the Tobacco Control Working Group.

Academia plays another key role in providing evidence to policy makers in support of decision making. Research institutes involved in the Tobacco Control Working Group include Hanoi School of Public Health, Social Institute, Institute of Economics, Academy of Finance and University of Trade [32]. The Prime Minister's Research Commission has an advisory role, but the limited number of technical advisors who are able to provide direct support to the government constrains the potential of technical leadership in Vietnam [35]:

"Governments in foreign countries have big consulting networks. The government of USA has over 6,000 consulting firms. Our government has only a research team. Apart from the government office, there are only a few research institutes under the ministries." (*GO*; quote from interview)

### Tobacco industry

The tobacco industry in Vietnam is largely state-owned. VINATABA stands at the heart of tobacco industry, manufacturing 46.2 billion sticks of cigarettes which covered 58% of the domestic market share in 2007 [36,37]. The production capacity of cigarettes in Vietnam is 90-100 billion sticks per annum, which are either domestically consumed or exported to other countries [38]. Tobacco industry ranks third, after oil and alcohol, among the major contributors to the government revenue in Vietnam (*MTI *and *MOH*; interviews). The tobacco industry is known as one of the most powerful marketers and lobbyists.

"For the government of Vietnam, there are two main sources of information for tobacco: MOH; and tobacco companies." (*NAO*; quote from interview)

"I think that, very likely, some barriers come from the tobacco industry, I'm sure. ...they are excellent lobbyists. So I think that one of the first difficulties must be coming from the tobacco industry." (*HBC*; quote from interview)

Transnational tobacco companies are increasing their shares on foreign brand tobacco products, which are domestically produced under licensing contract and joint venture agreements. Currently there are three foreign companies distributing tobacco products in Vietnam: British American Tobacco; Phillip Morris International; and Japan Tobacco Inc. In 2005 about 25% of cigarettes sold in Vietnam were linked to foreign brands. This has increased to nearly 30% in 2008 [39]. Lee et al. [40] discussed the strong political influence exercised by British American Tobacco to gain access to the Vietnamese market. The presence of transnational tobacco companies within the tobacco policy is manifested in the Decision 88/2007/QD-TTg on the strategy of tobacco industry [41] where the above three enterprises are quoted as "strategic partners" in producing cigarettes. The number of foreign tobacco enterprises may increase with the recent movement of Common Effective Preferential Tariff negotiated among member states of the ASEAN, which has largely abolished tariffs on tobacco leaf imports, and other non-tariff barriers will be removed in the future [42]. The Thai Tobacco Monopoly has initiated engaging with a joint venture with a private company to market its product in Vietnam [42]. These advancements of foreign companies in Vietnam contradict the principle of the National Tobacco Control Policy that proscribed new investments exceeding the production capacity of tobacco above the level of 2000 [8]. Although not explicitly raised at interviews, such encroachments clearly suggest that transnational tobacco companies certainly form part of the influential actors in Vietnam.

## Discussion

Grindle and Thomas [13] distinguish between "crisis" and "politics-as-usual" models of decision making, and postulate different hypotheses for each. In "crisis" the level of the decision-making hierarchy moves upwards, pushed by high stakes for political stability, while under "politics-as-usual" the issues are more relaxed, with decision making focused on bureaucratic power relationships, and with bureaucratic agencies competing with each other to win support or challenge opposition. With tobacco a largely state-owned enterprise, directly linked to both MTI and MOF, and providing predictable government revenue, the views outside the health sector are largely dominated by "politics-as-usual" and a focus on the short term economic returns from the industry.

"One of the elements which slow down the legislation process of tobacco control law is the [competing] priorities within the government: tobacco is not a hot topic." (*MOH-TE*; quote from interview)

In contrast, the tobacco issue is seen more as "crisis" by MOH and other advocates for tobacco control, as they perceive its increasing morbidity and mortality, and calculated the projected health and economic effects of current smoking rates over the next decades.

Comparing the position of tobacco and HIV/AIDS policies, both of which risk significant mortality unless controlled, provides some useful insights. Both these two policy environments rest upon a strong government apparatus, with central state organs dominating the policy dialogue, although limited windows are made open to civil society. However, the levels at which decisions have been made differ for the two policies. The drastic reform and rapid change of the HIV/AIDS policy were pursued by the Communist Party with determination, driven by the high political stakes arising from the perceived threat of HIV/AIDS to the nation's development--"crisis" in policy terms [15]. This is in clear contrast to the tobacco control policy, where the main drivers are at the bureaucratic level, with internal ambivalence in the positions of different stakeholders within the crucial Party organs and relevant ministries.

Table 3 interprets the positions of each actor to the Tobacco Harm Prevention Law as was assessed by the researchers during interviews and focus group discussion. While respondents in both interviews and focus groups were careful not to express overt opposition to the proposed Tobacco Harm Prevention Law, their perceptions on tobacco control could be grouped in several categories. Some may appreciate the importance and fully support tobacco control interventions, while others may show supportive attitude yet are sceptical with the total benefit of tobacco control to the society.

**Table 3:** Interviewees' positions on tobacco issues

Stakeholder and cluster	**Tobacco as a public health priority**^**a**^	**Tobacco as a socio- economic issue**^**b**^	**Position to tobacco control policy**^**c**^
**Key policy decision makers**			
*NAO*	++	++	+++
*DPT*	+	++	++
**The government**			
*GO*	++	++	++
*MOH-TE*	+++	++	+++
*MOH-L*	+++	+++	+++
*VINACOSH*	+++	++	+++
*MTI*	++	+++	+
*MOF*	++	+++	+
**Tobacco control advocates**			
*VPHA*	+++	+	+++
*WHO*	+++	+	+++
*HBC*	+++	+	+++

NB: The abbreviations in Italics represent the informants as defined in Table 2.

^a ^+++ High priority ++ Unclear/need more information + Not a high priority

^b ^+++ Major issue ++ Unclear/need more information + Not a major issue

^c ^+++ Supportive ++ Unclear/need more information + Reluctant

The relative weights on health and socioeconomic issues placed by each stakeholder determine the individual position on tobacco control. Ministries are divided into sectors, which makes their positions relatively clearer, while those at the higher hierarchy are trying to balance these competing agendas manifested in their ambiguous positions. However, as the position of MOF indicates, economic issues seem to have more immediate influence in setting policy directions, with the consequences of health gains less visible, and perceived as relegated to a distant future. But this in turn suggests that the provision of evidence on socioeconomic implications may influence the positions of some stakeholders on tobacco control. In Table 3, the overall stakeholders' position to tobacco control policy would shift substantially in favour of tobacco control only if the economic framing of the issues were challenged. In this sense, the current balance between the two competing issues: tobacco as a public health priority issue; and tobacco as a socioeconomic issue, needs to be challenged. Policy makers need to see in economic terms the impact of tobacco as a health priority--with evidence that underscores both the magnitude of the latent macroeconomic threat and the scale of that impact, both currently and for a generation to come [43]. (This should not be confused with the commonly made claims of future saving of healthcare costs due to reduced smoking, which was argued by Barendregt et al. [44].)

In the conduct of this research, researchers have discussed with tobacco control advocates their strategies, noting that they are acutely aware of what might, or might not, be acceptable to the most stakeholders, and modify their positions accordingly. Interestingly, health advocates are acutely aware about the opposition they face, at times rehearsing apologist positions for the industry, arguing that "health staff need to understand the full picture". Despite the demonstrable cost-effectiveness of increasing taxation [45], for example, caution was common:

"I think, to increase tax, it should be 2-3 years later because we have just increased tax in early 2008." (*VINACOSH*; quote from interview)

The FCTC action plan recommends that a roadmap should be developed for excise tax increases, but does not provide any target level of percentage to which the tax rate should be increased [9]. Further, concrete requirements for the excise tax increase are not included in the draft Tobacco Harm Prevention Law, with advocates attributing its exclusion to the fact that excise tax is managed by MTI, though precedents exist in other decrees and decisions managed under different ministries. Although there may be concessions among the advocates regarding the uncertainties surrounding the broader socioeconomic implications, there is a sense of bargaining to win those priority issues which are more likely to be accepted over the less likely ones.

Finally, this study has some limitations that are worth noting. We used the approach proposed by Varvasovszky and Brugha [12] as the guide for our analysis. However, the approach itself is not without limitations. First, a major drawback rests with its cross-sectional nature while analysing a policy environment that is often subject to rapid changes. Such changes may originate from internal events, external events, and even from the stakeholder analysis itself [12]. Therefore, the validity of findings from the analysis may diminish fairly quickly if a major event takes place (e.g. changes in leadership). Further, the validity and reliability of responses can be difficult to establish. The individuals interviewed may not accurately reflect the views of their host organisations depending on the degree of stability of positions the respondents assume. Although this drawback can be minimised by triangulating findings from different sources, certain levels of uncertainty remain.

Another limitation is the absence of interviews with representatives from the tobacco industry, including VINATABA and transnational tobacco companies with which it has established licensure or joint venture agreement. Access to tobacco industry spokespersons for policy research is generally recognised as difficult, as researchers in other countries confirm [46,47]. However, because of government ownership of tobacco industry in Vietnam, industry positions advocated in negotiations with VINACOSH were able to be confirmed, to a certain extent, in discussions with their responsible government agency, the MTI.

## Conclusion

The fundamental paradox surrounding the tobacco control policy in Vietnam arises from the contradictory position of a government that benefits from manufacturing of tobacco products, and is also responsible for controlling tobacco consumption. The short-term economic interests of the ministries responsible for the largely state-owned tobacco industry remain in tension with the MOH, and health advocates who recognise the long term implications of what is one of the major risk factors in the burden of disease. The industry's importance is reflected in the ambivalent position presented by high-ranking decision makers with respect to Tobacco Harm Prevention Law. Such dilemmas are further exacerbated by the one party governance, where bureaucrats do not have the tradition to express overt oppositions. This is revealed in the somewhat hesitant position of the MOH to strongly insist for a further tax increase. The perceptions of negative impacts on government revenue and the macro-economy, coupled with the reluctance of MOH to challenge these issues from health perspective too directly, means that tobacco control policy has yet to be recognised as "crisis" in policy term. The overall policy environment will shift in favour of tobacco control only if the economic framing can be challenged.

## Competing interests

The authors declare that they have no competing interests.

## Authors' contributions

All authors participated in the conception and analysis of the study. HH, TAK and ADN conducted and transcribed interviews and focus group discussion. PSH provided technical expertise for the whole process. HH drafted the initial manuscript and all authors gave comments, edited and approved the final manuscript.

## Supplementary Material

Additional file 1 **Questionnaire form**. Survey questionnaire form distributed to members of the National Assembly. Click here for file
