# Supplementary material for: The development of Tobacco Harm Prevention Law in Vietnam: stakeholder tensions over tobacco control legislation in a state owned industry
Source: Subst Abuse Treat Prev Policy. 2011 Sep 18;6:24. doi: 10.1186/1747-597X-6-24 (PMC3191345; doi:10.1186/1747-597X-6-24)
Supplement: Additional file 1 — Questionnaire form. Survey questionnaire form distributed to members of the National Assembly. [file 1747-597X-6-24-S1.PDF]

QUESTIONNAIRRE TO EXPLORE OPPINIONS OF NATIONAL ASSEMBLY ON SOME ISSUES RELATED TO REGULATIONS IN THE DRAF LAW ON THE PREVENTION AND CONTROL TOBACCO HARMS

In order to explore the needs for evidence and identify priority policy issues for research to serve as a basis to formulate and approve the Law on prevention and control of tobacco harms, the HSPI, in coorperation with Department of Treatment, Ministry of Health, is asking for opinions of the National Assembly members and representative agent of a member of the National Assembly on some issues related to regulations in the draft Law on the Prevention and Control of Tobacco harms.

Please kindly pay attention to and provide information by filling in this questionnairre!

(please check √ as appropriate)

----

1. What is your position?
    a. A member of the National Assembly
    b. Representative agent of a member of the National Assembly
    c. Other, detail………………………………………………………………………….
2. What level are you working for?(central, provincial, district, commune)………………………..
3. What is your usual occupation?:.....................................
4. Have you ever heard/read the Draft law on prevention and control of tobacco harms?
    a. Yes b. No
5. Where did you hear from?
    a. Ministry of Health
    b. National Assembly
    c. Other
6. Have you ever discussed the Draft Law on prevention and control of tobacco harms?
    a. Yes b. No

***Section A. How effective do you think the following policy options would be in decreasing smoking habit* across population?**

Statement	*Very effective*	*Effective*	*Not effective*	*Could increase smoking*	*Unsure*
1.Carry out effective campaigns about the harmful effect of smoke on mass media					
2.Print big photographic and text health warnings on the package,covering at least 50% its area					
3. Issue and effectively implement the ban or limited usage of smoking images on movies and other forms of performances					
4. Issue and effectively implement the prohibition of smoking within all indoor offices (office with roof and/or surrounded walls), public indoor spaces and public transportation means.					
5.Prohibit all kinds of sponsor from tobacco companies					
6.Increase special consumption tax on tobacco products					
7. Impose a tax or fee (1-2% of retail price of tobacco products) to set up National Fund for Health Improvement and Tobacco Harms Control					

Section B: In your opinion, how available are the scientific evidences of the prevention and control of tobacco harms?

Thera are firm scientific evidences that smoking is harmful to community's health and socioeconomic. Please rating the following statements upon your agreement

Statement	*Agree*	*Disagree*	*Needs more evidences*	*Unsure*
1. Smoking causes many serious diseases such as cancer, heart and lung diseases, and results in death				
2. Passive smoking (breath in smoke from other smoker) causes diseases and death				
3. Social - cost from smoking diseases outweighs tobaco's economic benefit				
4. Increase tax on tobacco products can reduce consumption				
5. Increased tobacco's tax will raise state revenue in spite of smuggling.				
6. To reduce smoking, photographic warnings (print on tobacco package) are more effective than texts warnings				
7. Promulgate on mass media about the harmful effects of smoke will reduce smoking rate.				
8. Number of deaths due to smoking is higher than the total number of dealths due to HIV/AIDS, TB and malaria.				
9. Small package (less than 20 sticks) will increase smoking in adolescent				

Section C. To prevent and control tobacco harms, do you support the following policy options?

	Totally support	*Support but need more evidence*	*Will not support until evidence is showned*	*Will not support in any cases*
1. Print photographic and text health warnings on cigarette package				
2. Restrict all kinds of sponsor from tobacco companies				
3. Issue and effectively implement the prohibition of smoking within all indoor offices (office with roof and/or surrounded walls), public indoor spaces and public transportation means.				
4. Increase tax on tobacco products				
5. Impose a tax or fee (1-2% of retail price of tobacco products) to set up National Fund for Health Improvement and Tobacco Harms Control				

Section D: What further evidence do you need to support the Draft Law on Prevention and Control Tobacco Harms?

1. Harmful effects of smoking on health and socioeconomic?
   ....
2. Treatment cost of smoking diseases?
   ....
3. Increase tax on tobacco products
   ....
4. Effect of mass media campaigns on the prevention and control of tobacco harms
   ....
5. National Fund for Health Improvement and Tobacco Harms Control
   ....
6. Other, (please detail)
   ....
   ……………………………………………………………………………………………..
   ……………………………………………………………………………………………..
   ……………………………………………………………………………………………..
7. If possible, please indicate whether you are a smoker or not?
   a) Yes b) No

Thank you very much
